# TACkling Cancer by Targeting Selective Protein Degradation

**DOI:** 10.3390/pharmaceutics15102442

**Published:** 2023-10-10

**Authors:** María del Mar Noblejas-López, David Tébar-García, Raquel López-Rosa, Ana Alcaraz-Sanabria, Pablo Cristóbal-Cueto, Alejandro Pinedo-Serrano, Lorenzo Rivas-García, Eva M. Galán-Moya

**Affiliations:** 1 Centro Regional de Investigaciones Biomédicas (CRIB), Campus de Albacete, Universidad de Castilla-La Mancha, 02008 Albacete, Spain; mariadelmar.noblejas@uclm.es (M.d.M.N.-L.); david.tebar2@alu.uclm.es (D.T.-G.); raquel.lrosa@uclm.es (R.L.-R.); analucia_alcaraz@hotmail.com (A.A.-S.); pablocristobal09@icloud.com (P.C.-C.); alejandro.pinedo@uclm.es (A.P.-S.); 2 Unidad de Investigación, Complejo Hospitalario Universitario de Albacete, 02008 Albacete, Spain; 3 Facultad de Enfermería, Campus de Albacete, Universidad de Castilla-La Mancha, 02006 Albacete, Spain

**Keywords:** protein degrader, PROTAC, PHOTAC, CLIPTAC, AUTAC, ATTEC, LYTAC, DUBTAC, precision medicine, nanomedicine

## Abstract

Targeted protein degradation has emerged as an alternative therapy against cancer, offering several advantages over traditional inhibitors. The new degrader drugs provide different therapeutic strategies: they could cross the phospholipid bilayer membrane by the addition of specific moieties to extracellular proteins. On the other hand, they could efficiently improve the degradation process by the generation of a ternary complex structure of an E3 ligase. Herein, we review the current trends in the use of TAC-based technologies (TACnologies), such as PROteolysis TArgeting Chimeras (PROTAC), PHOtochemically TArgeting Chimeras (PHOTAC), CLIck-formed Proteolysis TArgeting Chimeras (CLIPTAC), AUtophagy TArgeting Chimeras (AUTAC), AuTophagosome TEthering Compounds (ATTEC), LYsosome-TArgeting Chimeras (LYTAC), and DeUBiquitinase TArgeting Chimeras (DUBTAC), in experimental development and their progress towards clinical applications.

## 1. Introduction

Cancer remains the second leading cause of death worldwide; in fact, approximately one in six deaths are caused by this disease. Currently, cancer incidence is growing globally and causing physical and emotional damage, as well as financial strain [1]. Current therapies are based on chemotherapy, radiotherapy, and surgery; however, these treatments are associated with undesirable effects such as neurotoxicity or hepatotoxicity and could develop high rates of resistance; thus, looking for new strategies for cancer treatment is a focus of all biomedical sciences. In that way, targeted therapies have emerged as a new tool for anticancer drug development [2]. These therapeutic agents provide novel chemical compounds in their structure that enhance specific interactions with target-selective proteins, promoting their degradation and reducing side effects associated with conventional treatments [3]. Hormone therapies, signal transduction inhibitors, gene expression modulators, apoptosis inducers, angiogenesis inhibitors, and immunotherapies are some examples of targeted therapies approved in clinical settings. However, certain side effects are associated with this type of therapy, such as thrombocytopenia, dermatological toxicities (e.g., erythema), fatigue, and several cardiovascular alterations (e.g., hypotension and thromboembolism) [4,5,6]. In addition, intracellular proteins are often undruggable (due to the loss of some specific cell surface proteins) and as a result, cancer cells that rely on them could evade the classical treatments of chemotherapy and radiotherapy. In this line, targeted protein degradation (TPD) is gaining momentum in cancer therapy, as it can not only target undruggable proteins but also overcome cancer resistance and avoid undesirable side effects. Thus, small-molecule degraders have emerged as novel therapeutic options [7].

In mammalian cells, where the protein degradation process is essential for maintaining cellular homeostasis, degradation is mainly mediated by the ubiquitin-proteasome pathway, which begins with the union of an E3 ubiquitin ligase to a specific substrate, leading to degradation via the proteasome [8]. This is the basis of the development of molecular glues and PROteolysis Targeting Chimeras (PROTACs), many of which are currently under clinical validation. However, there are other pathways for protein degradation, such as the endosomal-lysosomal system or the autophagy-lysosome pathway, which are being exploited for the design of protein-degrading tools. In the former, the fusion of endosomes and lysosomes forms a membrane bilayer that generates an acidic space optimal for the activation of hydrolytic enzymes, including proteases, nucleases, and lipases, that are able to degrade proteins [9]. Accordingly, LYsosome-TArgeting Chimeras (LYTACs) use lysosome-targeting receptors to complete targeted protein degradation inside lysosomes. In contrast with PROTACs, LYTACs can efficiently target extracellular and transmembrane proteins. In the latter, the autophagy-lysosomal pathway, intracellular macromolecules, long-lived proteins or aggregates, and cytoplasmic organelles are digested by the fusion of the autophagosome and lysosome, which contains degradative enzymes. The degraded intracellular and extracellular material is recycled through autophagy and endocytosis to supply energy to cells [10]. In this line, AUtophagy-TArgeting Chimeras (AUTACs) aim to promote the degradation of target proteins via autophagosomes. Recently, a new class of molecules aiming to target protein stabilization by eluding the ubiquitin-proteasome degradation pathway has emerged. DeUBiquitinase-TArgeting Chimeras (DUBTACs) mediate the recruitment of a deubiquitinase to avoid the degradation of the protein of interest (POI) via the proteasome. A summary of the therapeutic pathways used by the abovementioned TACnologies and their derivates is described in Table 1.

In this review, we summarize the current applications of protein-degrading tools and summarize the first two decades of PROTAC development and the status of clinical translation of TPD. We also focus on discussing key questions relevant to what TACnologies could achieve therapeutically and what is needed to move the field forward over the next 20 years.

### A Brief History of TPD

Despite its relatively short history, TPD has gained a great amount of interest in recent years, especially in the cancer research field.

Following the discovery of the ubiquitin-proteasome degradation system in the 1990s, TPD technologies began to emerge. In the early nineties, cyclosporin A, rapamycin, and FK506 were identified as the first “molecular glues” [11,12]. Molecular glues are proximity-inducible small molecules that favor protein-protein interactions, promoting the dimerization or colocalization of two or more proteins, which inactivates one of them or a third player.

**Figure 1:**
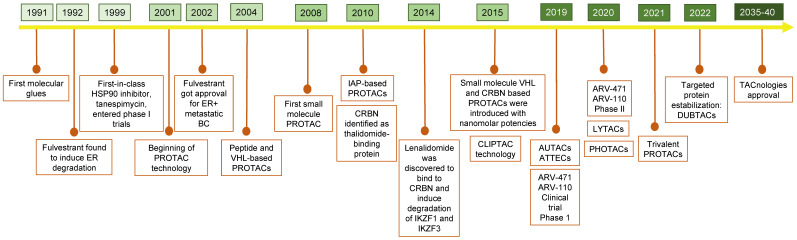
Timeline of the past, present, and future of the TAC-based technologies.

Next, in 1992, Fulvestrant^®^ and Tanespimycin^®^, considered the pioneer alternatives for TPD, were described. Fulvestrant^®^ is a downregulator of the estrogen receptor that induces a conformational change that leads to its target protein degradation [13]. It was first approved by the Food and Drug Administration (FDA) in 2002 for the treatment of metastatic breast cancer in postmenopausal women [14]. Then, between 2001 and 2004, the development of PROTAC technology emerged, and subsequently, the use of new trends for drug design (i.e., the inclusion of small molecules or peptides with the ligase VHL) increased the efficiency against target proteins [15]. From 2008–2015, new ligases were developed into PROTACs, such as MDM2, IAP, CRBN, and VHL E3 ubiquitin ligases. Later, optical control for protein degradation and light-inducible Photochemically targeted chimeras (PHOTACs) were designed [16]. The most recent degrader agent was developed in 2021 (Trivalent PROTAC). It was designed with higher binding strength against more than one receptor in the POI improving the degradation efficiency and reducing associated details, exemplifying the potential of this type of drug. Figure 1 shows the timeline sequence of targeting protein drug development, and Table 2 describes some examples of their use.

## 2. PROTACs: PRoteolysis TArgeting Chimeras

**Figure 2:**
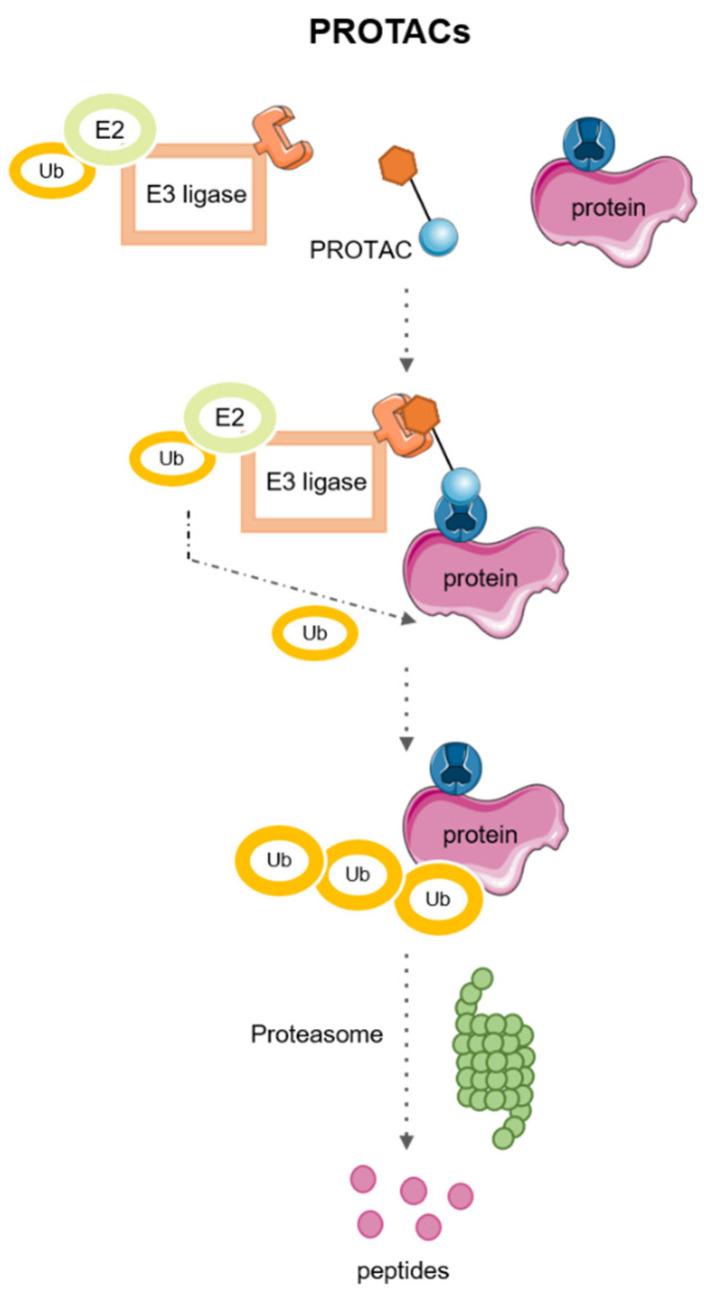
Mechanism of action of PROTACs.

PROteolysis TArgeting Chimeras (PROTACs) are heterobifunctional molecules assembled by two ligands linked by a linker. One of the ligands recognizes the POI, and the other recognizes an E3 ligase. The proximity between the POI and E3 ligase results in ubiquitination and subsequent degradation of the POI by the ubiquitin–proteasome system (UPS) [28]. Figure 2 shows the mechanism of action of PROTACs.

The concept of PROTAC emerged more than 20 years ago, specifically in 2001. This technology has gained interest during the last few years, exposing novel drugs for cancer treatments [29]. The literature based on PROTACs has greatly increased in the recent years, capturing the attention of the scientific community. PROTAC-DB is a free online database that provides information on new PROTACs, POIs, E3 ligands and linkers [30]. In addition, several PROTACs are currently being evaluated for cancer treatment in clinical trials, and this information is summarized in Table 3.

The main advantages of PROTACs related to traditional small molecule inhibitors include the noncatalytic nature of the target, reduced dosage and dosing frequency, more potent and longer-lasting effects, greater selectivity to reduce potential toxicity, efficacy against drug resistance mechanisms, and expanded target space including scaffold proteins [31].

### 2.1. PROTACs: Examples of Applications

#### 2.1.1. BCL-2 Protein Family

Some aspects of tumor metabolism such as the overexpression of BCL1 and BCL2 play an important role not only in tumor initiation and progression but also in the development of resistance and the evasion of apoptosis [31]. The use of inhibitors against BCL proteins has shown some therapeutic limitations related to the toxicity associated with these drugs in patients with solid tumors, such as small-cell lung cancer, metastatic melanoma, lung cancer, prostate cancer, squamous cell carcinoma of the head and neck, brain and central nervous system tumors [32].

PROTACs have emerged as a new tool for overcoming these limitations; for example, a BCL-xl/2 dual inhibitor agent (Navitoclax-ABT-263) induced thrombocytopenia in patients. Thus, its use has not been approved in clinical therapy [33]. However, a new drug (DT2216) was synthesized by several modifications of ABT263 and was included in a VHL-recruiting PROTAC that demonstrated their capacity for linking to BCL-xL/2; however, this new drug did not report capacity for linking to the BCL2 isoform, and consequently, this PROTAC was not used in BCL2-dependent T-cell acute lymphoblastic leukemia and T-cell lymphoma. Other authors combined with ABT199 (Bcl2 selective inhibitor) to overcome these problems [34].

On the other hand, other authors described PROTACs that can degrade both targets (BCL-xL/2) as 753b. This drug promoted a higher expression of MCL-1 in cell lines mediated by the induction of cell death and the elimination of senescent leukemia cells. Thus, in vivo experiments have shown higher efficiency when combined with the chemotherapeutic agent cytarabine in patient-derived xenografts [35].

#### 2.1.2. Cycling Dependent Kinases

Cyclin-dependent kinases (CDKs) are a large family of proteins implicated in cell cycle and transcriptional regulation. CDKs are attractive targets for the development of small-molecule chemical inhibitors. It is noteworthy that some of these compounds have reached the clinical setting, such as those acting on CDK4/6 [36,37]. 

PROTACs have several advantages compared to traditional inhibitors, i.e., PROTACs have demonstrated higher efficiency and selectivity against target proteins and overcome acquired resistance to traditional chemotherapeutic agents [38,39].

#### 2.1.3. Mitogen-Activated Protein Kinases

The mitogen-activated protein kinases (MAPK) include ERK, p38, and JNK MAPK subfamilies that control several intracellular mechanisms such as cell pathology impacts on cell proliferation or growth (ERK) and cell death (p38 and JNK) [40]. 

The protein SHP-2, a non-receptor tyrosine phosphatase, that acts upstream of RAS-ERK, PI3K-AKT, and JAK-STAT can overcome downstream oncogenic signaling by using PROTACs against the ligand to disrupt its interaction with the receptor [41]. 

Drugs against BRAFV600E have improved efficacy in the clinical setting but their application has no durable effect due to the acquisition of resistance. The PROTAC P4B has shown higher target specificity against the target protein in melanoma cell lines than traditional inhibitors [42]. 

Selective PROTACs against the different p38 MAPK isoforms (α,δ) have been developed using a single warhead ligand against foretinib and another against the E3 link by VHL ligase; its efficiency and selectivity were similar for both isoforms [43]. 

The substoichiometric activity of irreversible PROTACs has been shown to be less effective than that of covalent-reversible PROTACs, as is the case of YF135, the first enabled to bind to VHL ligase to mediate KRASG12C degradation via ubiquitin-proteasome [44].

### 2.2. Trivalent PROTACs

Novel trivalent PROTACs work more efficiently than traditional bivalents due to the combination of two physical properties: affinity and avidity; both being measures of binding strength. While affinity refers to the binding strength at a single binding site, avidity is a measure of total binding strength [45]. Complex avidity is influenced by several factors, mainly cooperativity and valency; positive cooperativity (α > 1) between the E3 ligase and the target protein allows both to establish efficient interactions, leading to the formation of functional ternary complex [46]. 

**Figure 3:**
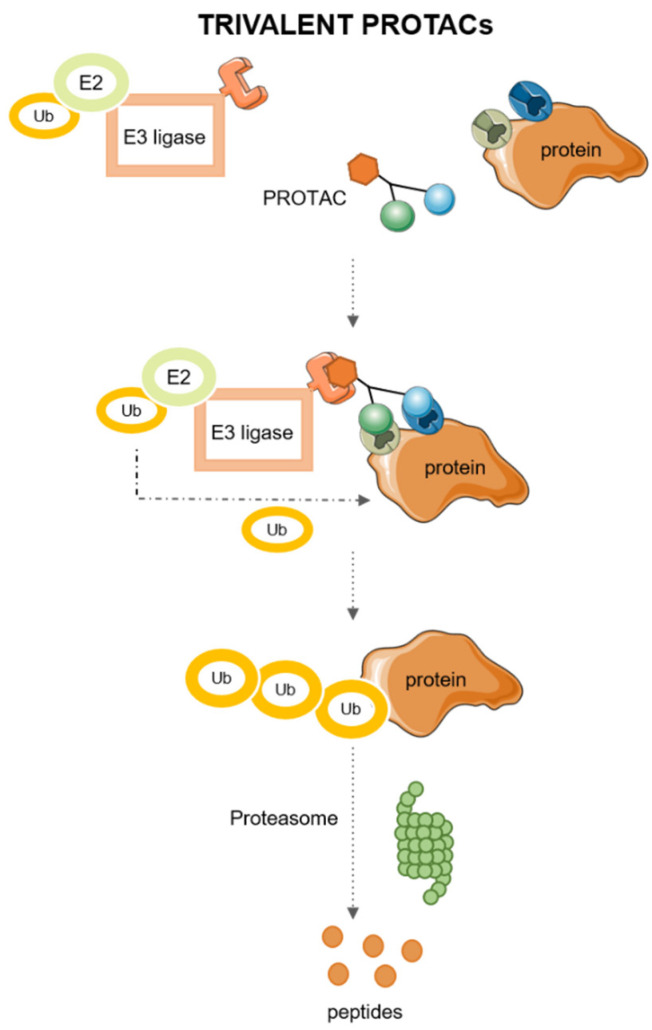
Mechanism of action of Trivalent PROTACs.

Trivalent PROTACs have higher binding valency than traditional PROTACs, as they carry two recognition ligands for the same POI in addition to the E3 ligase binding ligand, which allows the E2 to recruit ubiquitin molecules and transfer them to the POI for further degradation via the proteasome more efficiently than bivalent ones [26]. The mechanism of action of trivalent PROTACs is described in Figure 3.

## 3. Modulating the Activation by Light: PHOtochemically TArgeting Chimeras (PHOTACs)

**Figure 4:**
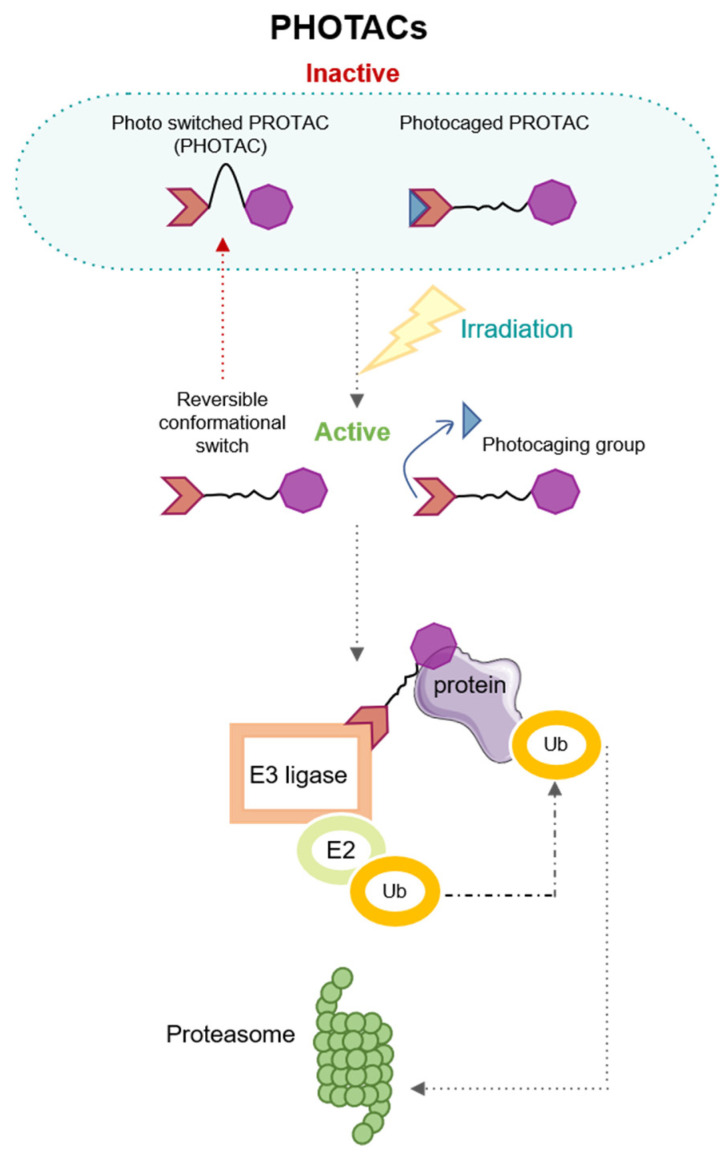
PHOTACs strategy of action.

Past pieces of evidence have related the use of light to medicine; in fact, in ancient Egypt, some diseases, such as psoriasis or vitiligo were treated employing light and vegetal-derived remedies [47]. Currently, light irradiation is a novel tool for precision cancer therapies; thus, light could be directed to cell-inducing physiological changes promoting endogenous biochemical reactions. However, light irradiation could adjust changes in therapeutic cancer agents increasing their efficiency, in that way, light irradiation could produce changes in the structure of gold nanoparticles (photodynamic therapy) [48] or could generate heat by the electronic oscillation (photothermal therapy) when nanoparticles are irradiated by visible light [49]. Ongoing trends postulate that the degradation of proteins could be controlled by Photochemically targeted chimeras (PHOTACs); these therapeutic agents are formed by an E3 ligand, a light switch, and a specific ligand of the target protein. Their functional strategy is described in Figure 4.

Within the photochemical groups, there are two types: photocaged and photo-switches. Photocage would be irreversibly activated by light, leaving PROTAC active, and would have to be deactivated by metabolism. In contrast, photo-switches are activated reversibly, adding an extra level of control [50].

The first group to describe the incorporation of small photocaged clusters of auxin into PROTACs was Gautier and colleagues [51]. They were followed by groups such as Li and collaborators who described the incorporation of a photocaged group formed by a nitroveratryloxycarbonyl group on the glutarimide nitrogen of the origin molecule obtaining a new photodynamic molecule named opto-pomalidomide. These authors demonstrated the efficiency of this PHOTAC in inducing the ubiquitination of IKZF1/3 by CRBN in myeloma cells in a dose-dependent and irradiation-dependent manner [22].

In addition, Reynders. M. and co-workers included azobenzene photo-switches in a CRBN ligand and showed degradation activity of BRD2-4 and FKBP12 proteins via their union with the CRL4CRBN complex. Furthermore, this PROTAC reported modulable activity and visible light (380–440 nm), and no activity in dark conditions [25]. This fact could help to induce new therapeutic tools mediated by light irradiation and could enhance the implementation of personalized medicine.

The main advantages of PHOTACs are incorporating photo-switches into PROTACs to control their activation with blue-violet wavelengths and minimizing off-target effects and toxicity. However, this approximation has shown several disadvantages, such as radiation exposure or the inability to target several proteins [25,50].

## 4. Application of Click Chemistry for PROTACs: CLIPTACs

Currently, the design trends of PROTACs include two ligands in the same drug. This fact generates molecules of high molecular weight, which on many occasions prevents their entry into the cell, altering some pharmacokinetic aspects. To solve these problems, it has been possible to resort to click chemistry, a field of chemistry initiated by Sharpless that tries to develop a faster and more specific efficiency for some chemical reactions. This way, Heightman’s group developed a new technology for the synthesis of PROTACs, CLIPTACs (CLIck-formed Proteolysis TArgeting Chimeras), which are designed by the inclusion of two pro-drugs as precursors of PROTAC [27]. These precursors have no activity in the extracellular domain; then by an intracellular click reaction, both elements are activated. The CLIPTAC complexes are formed in the intracellular space and self-assembled to create a functional CLIPTAC by binding simultaneously to an E3 ligase and the target protein.

Hence, one of these pro-drugs needs to include a trans-cyclo-octene (TCO) and a tetrazine, to promote inverse electron-demand Diels-Alder reaction that will generate the definitive CLIPTAC [52,53,54].

**Figure 5:**
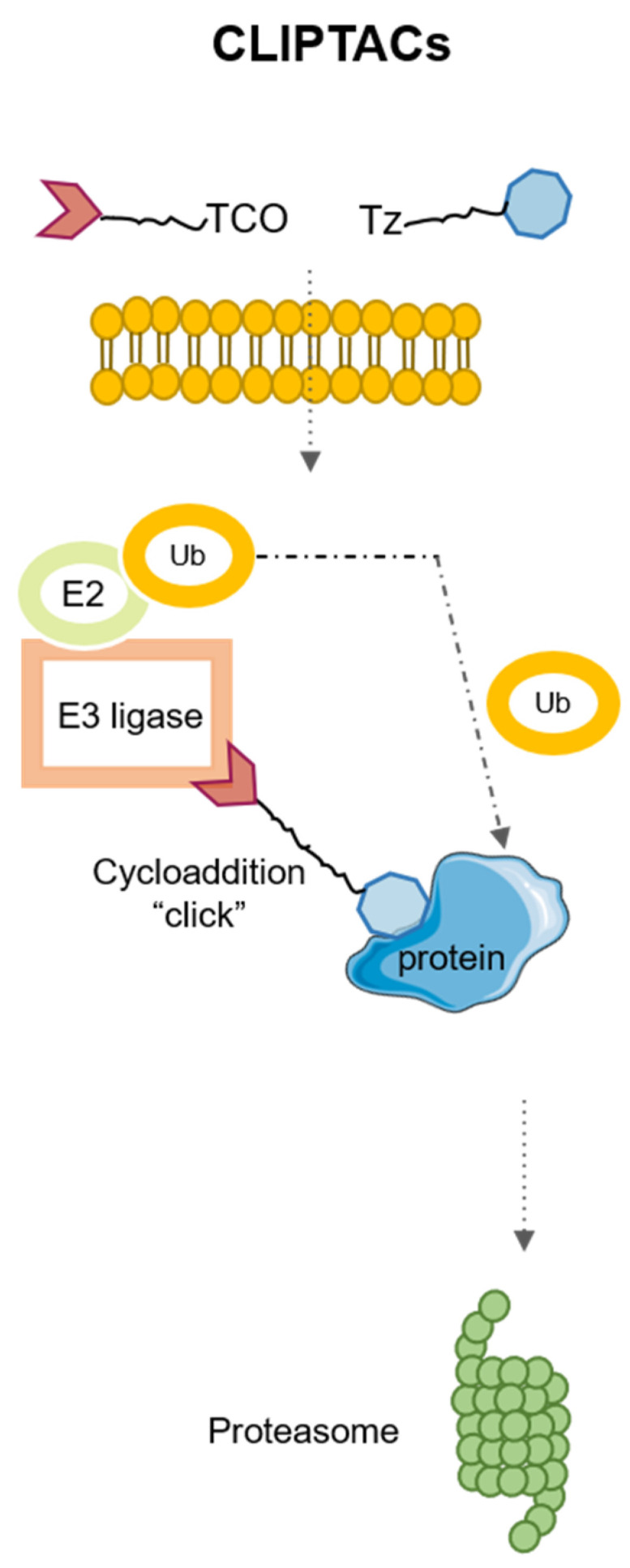
Description of the CLIPTAC therapeutic strategy.

The design of CRBN-based CLIPTACs consists of a tetrazine (Tz)-tagged thalidomide derivative that reacts with the TCO-labeled ligand of the target protein. The CLIPTAC mechanism is described in Figure 5. Heightman and colleagues reported efficiency of using CLIPTACs produced by a bio-orthogonal click combination of two precursors to degrade oncoproteins, such as BDR4 and ERK 1/2 in cancer cells in vitro. Moreover, they designed an efficient CLIPTAC system based on Tz-thalidomide and TCO-JQ1 and reported that that CLIPTAC did not promote protein degradation in the pre-assembled form; moreover, if the reaction occurred extracellularly, it did not achieve degradation either [27].

Recently, activity-based chemical probes (ABPs) have been used to design clicked-assembled PROTACs. Specifically, these new molecules have been synthesized using ABPs for the sirtuin family of proteins, more specifically SIRT2, which, similarly to other members of the family, contributes to cancer progression [55]. This CLIPTAC consists of an ABP probe conjugated to thalidomide 4′-ether-PEG2-alkyne, a CRBN recruiting ligand, using “click” technology. This CLIPTAC was able to efficiently inhibit SIRT2 in HEK293 cells at micromolar doses [56]. 

In conclusion, the intracellular click reaction could avoid the permeability problems motivated by the weight of traditional PROTACs. In addition, the use of new advances in chemistry as click chemistry could modulate and increase the efficiency of PROTACs.

## 5. AUtophagy TArgeting Chimeras (AUTACs) and AuTophagosome TEthering Compounds (ATTECs) 

Autophagy plays a conflicting role in cancer progression. On the one hand, in the early stages, autophagy maintains cellular quality control to reduce the production of reactive oxygen species, DNA damage, and defective cytosolic proteins, such as p62. The energy obtained from autophagy also decreases cellular glycolysis dependence and prevents the oncogenic transformation [57]. On the other hand, in advanced stages of cancer, autophagy helps cancer cells escape oxygen and nutrient deprivation, which promotes cell proliferation, decreases apoptosis, and favors the development of chemoresistance. This drug resistance is indeed mediated by the inactivation of pro-apoptotic factors and the activation of antiapoptotic effectors that promote survival signals [58].

**Figure 6:**
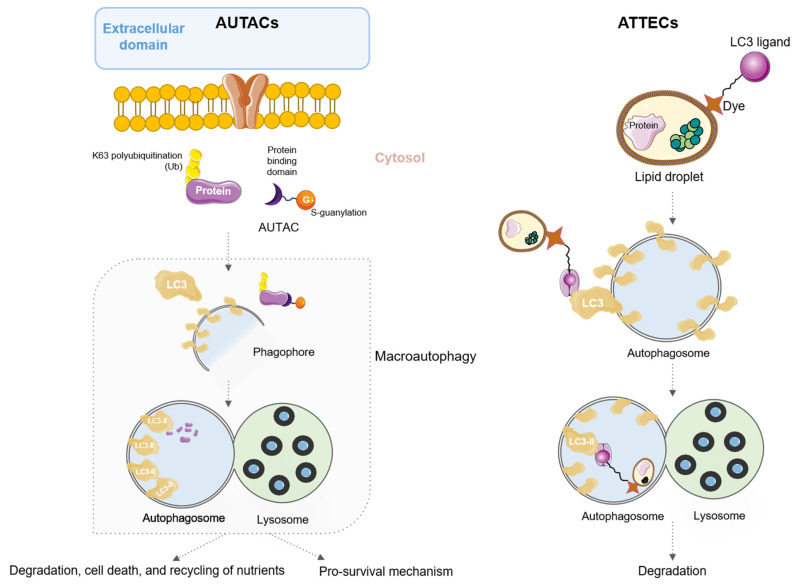
Description of the AUTAC and ATTEC therapeutic strategies.

Among PROTACs used for protein degradation, there is a group of new small molecules that use autophagy for this process, a group named AUtophagy TArgeting Chimeras (AUTACs). These molecules focus on the selective degradation of proteins and organelles by autophagy, a process that exists in cells for the maintenance of protein homeostasis and metabolic activities [59]. AUTACs are formed by a targeted protein binding domain and a tagging domain that induces degradation, joined by a linker. The tagging domain is usually an E3 ligase. The binding domain allows AUTAC attachment to specific substrates, whereas the tagging domain induces S-guanylation mediated by polyubiquitination of K63 to recognize autophagosome receptors. Once the autophagosome detects the protein to be degraded, it is phagocytosed and transferred to the lysosomes for degradation in the complex known as autophagolysosome (Figure 6) [60].

The first AUTAC described was published by Takahashi et al. and it was able to polyubiquitinate K63 on numerous substrates for recognition by the autophagosome receptor SQSTM1/p62, achieving successful protein degradation and mitochondrial turnover in Down syndrome (DS)-derived fibroblasts. Briefly, the authors reported that this AUTAC enhanced the removal of dysfunctional mitochondria, characteristic of DS patients, via mitophagy and increased mitochondrial biogenesis, therefore restoring mitochondrial homeostasis in those cells [61]. In addition, these authors described the efficiency of this AUTAC in a model of human cervical adenocarcinoma (HeLa cells). Later, Pei et al. published a potent AUTAC designed to degrade the BRD4 protein, a member of the bromodomain and extra-terminal domain (BET) family highly expressed in malignant solid tumors like breast cancer [62], in part due to its role in cancer stemness [63], by targeting the key autophagy protein LC3 [64]. The authors reported in vitro antitumor effects mediated by the ability of this AUTAC to degrade the BRD4 protein by triggering the autophagic process through targeted binding to LC3. They demonstrated efficacy in degrading BRD4 in multiple cell lines, including human cervical adenocarcinoma cells (HeLa), with greater than 90% efficacy, and several triple-negative breast cancer cells, such as MCF-7, MDA-MB-231, and MDA-MB-468, with degradation efficiencies ranging from 80% to 99% [64]. Furthermore, these authors confirmed that the antiproliferative effect of the synthesized AUTAC was indeed attributed to the modulation of autophagy, as demonstrated by comparison with a conventional autophagy inhibitor such as 3-methyladenine [64].

In certain diseases such as obesity or cancer, there is a significant accumulation of intracellular lipid particles (LD), which are not usually targeted by current therapies with PROTACS or AUTACs [65]. LD are intracellular organelles that store and release lipids that can be degraded by autophagy. Consequently, promoting specific lipid degradation could be an interesting approach to eliminating metabolic disorders in these diseases. For the treatment of these lipid particles, researchers have created bifunctional molecules proposed to hijack the autophagosomal pathway to potentially degrade any cellular component, named AuTophagosome TEthering Compounds (ATTECs). These molecules are formed by a domain that recruits LC3 proteins and the domain that joins LD, which is usually a Sudan dye, because of its high affinity for non-polar lipids [66]. Thus, ATTECs are able to degrade LDs by autophagy since they are made up of a monolayer of phospholipids, so other organelles whose membranes do not contain lipids are not affected by treatment with ATTECs [67]. In that way, ATTECs have been capable of clearing LD in cells derived from a murine model of hepatic lipidosis [67]. Moreover, recent studies have revealed novel strategies for degrading proteins and non-protein biomolecules by ATTECs using lipid droplets as targets [68]. Briefly, novel compounds interact with lipid droplets and the autophagosome protein LC3, promoting their degradation.

ATTECs are able to direct the autophagy process in protein and non-protein cellular components without the need for a tagging domain for autophagosome recognition, unlike PROTACs and AUTACs, as they are able to guide the formation of autophagosomes. However, the dyes used for the detection of non-polar lipids have a carcinogenic nature, so the development of new dyes or synthetic variants is needed to minimize off-target effects. Figure 6 shows a schematic summary representation of the mode of action.

## 6. LYTACs: LYsosome-TArgeting Chimeras

**Figure 7:**
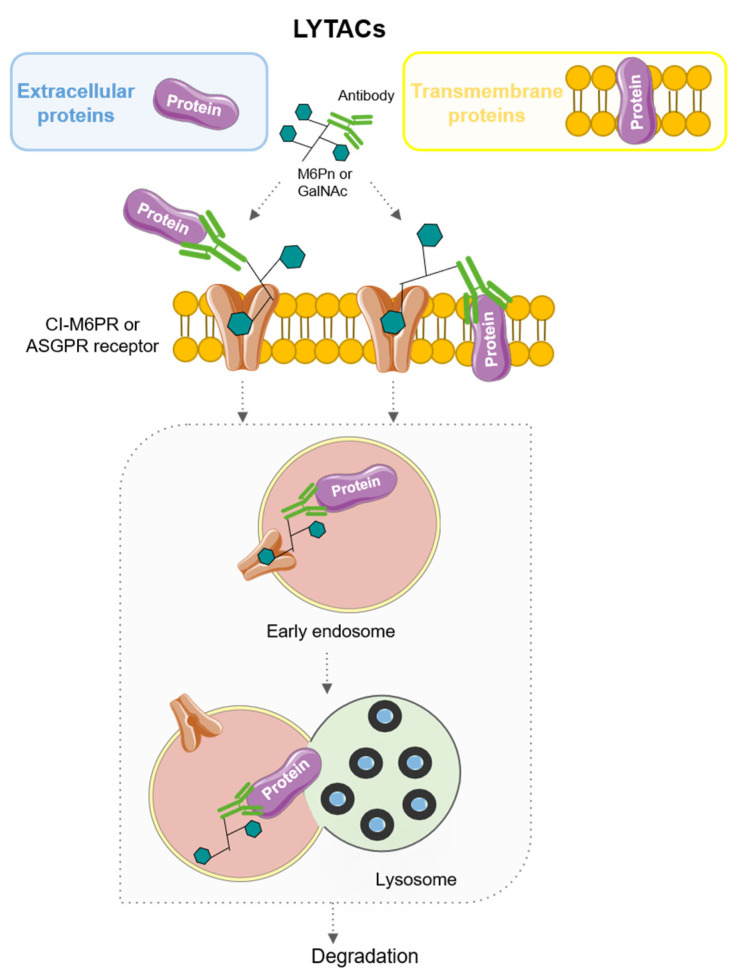
Mechanism of action of LYTACs.

LYsosome-TArgeting Chimeras (LYTACs) are bimodular molecules capable of targeting proteins for destruction in lysosomes. LYTACs, unlike the proteasomal pathway, are capable of binding to both lysosome-targeted receptors on the cell surface and extracellular or transmembrane proteins, via antibodies, to target them for degradation [69,70]. To date, mainly two receptors have been used. Their mode of action is described in Figure 7.

First-generation LYTACs use cation-independent mannose-6-phosphate receptor (CI-M6PR). Accordingly, the molecule is formed by an antibody, which interacts with the POI, and a synthetical glycopolypeptide, serine-*O*-mannose-6-phosphonate (M6Pn), which interacts with CI-M6PR, a receptor that binds to M6P-tagged proteins and transports them to lysosomes. Therefore, the POI indirectly binds CI-M6PR and is internalized by endocytosis, reaching lysosomes, where complete degradation occurs [69,71].

Second-generation LYTACs exploited the potential of a cell type-specific receptor, the asialoglycoprotein receptor (ASGPR), which is only expressed in hepatocytes, therefore exploring a tissue-specific approach for the design of these molecules [71,72]. These LYTACs can be conjugated to Galactosamine (Gal) or N-acetylgalactosamine (GalNAc) ligands. However, trivalent ligands (tri-GalNac) display a higher affinity for the receptor and therefore have shown better efficacy [71,72]. 

Several LYTACs have been developed to eliminate specific pro-tumoral targets. The epidermal growth factor receptor (EGFR, also known as HER1) is augmented in some solid tumors and its altered expression or mutation has been described as a tumor growth enhancer in different types of cancer, including ovarian cancer, glioblastoma and hepatocellular carcinoma (HCC) [73,74,75,76,77]. Under this premise, different types of LYTACs have proven efficient EGFR degradation in epithelial ovarian cancer and HCC cell lines using cetuximab, an EGFR-blocking antibody, as the POI ligand and conjugating it with M6Pn [69]. Ahn et al. also tested the targeting of EGFR with LYTAC. Their construct, which used GalNAc-tagged cetuximab, achieved more than a 70% reduction in EGFR protein levels in HCC cell lines [72].

Another tyrosine kinase member of the EGFR family, HER2, which is usually overexpressed in specific breast cancer subtypes and in some HCC, among other solid tumors [78,79], has also been targeted with novel LYTACs. Pertuzumab, an approved HER2 antibody by the FDA, has also been tagged with M6Pn in HCC in vitro models [72].

LYTACs may also display important effects in the tumor microenvironment by targeting cancer cell receptors involved in the cancer immune response. Banik et al. designed LYTACs able to target PD-L1, a driver of immune evasion in cancer that precludes T-cell recognition, therefore preventing tumor cell death [80]. Specifically, they conjugated anti-PD-L1 or atezolizumab, a PD-L1-blocking antibody, with M6Pn and tested their effects in the breast cancer cell line MBA-MB-231 and the Hodgkin’s lymphoma cell line HDLM-2, which expresses higher levels of CI-M6PR than the breast cancer model, respectively, observing a degradation of PD-L1 up to 70% [69].

Although most LYTACs use antibodies to bind their POI, GalNAc-LYTACs can be conjugated with molecules other than antibodies to destroy a specific target. Moreover, conjugation of a polyspecific integrin-binding peptide (peptide-based LYTACs) to a Gal-NAc ligand significantly reduces cell proliferation in HCC cell lines, showing long-term inhibition. This integrin-ablating LYTAC opens novel strategies for some tumors with abnormal integrin expression [72,81].

As mentioned before, PROTACs can degrade a specific protein blocking downstream signaling. However, this technology is restricted to intracellular targets. The use of LYTACs overcomes this obstacle, attaining a successful degradation of extracellular soluble or membrane proteins. Moreover, a tissue-dependent degradation using specific lysosomal receptors can be achieved. In this regard, further studies are needed in search of new receptors to develop new tissue-specific protein degradations, such as ASGPR in hepatic cells [72].

## 7. DeUBiquitinase-TArgeting Chimeras (DUBTACs)

**Figure 8:**
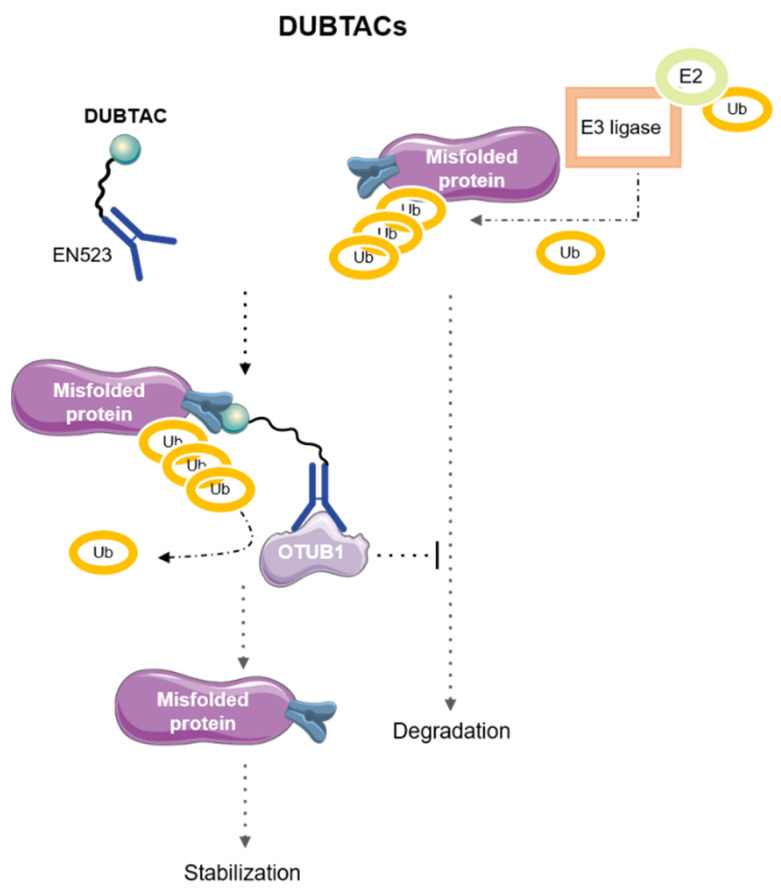
Description of DUBTACs’ therapeutic strategy.

Some diseases, such as Alzheimer’s disease or cancer, rather than systematically pursuing protein degradation for initiation or progression, can also be triggered or worsened by stabilization of a POI. In this line, a novel class of agents, DeUBiquitinase-TArgeting Chimeras (DUBTACs), which consist of heterobifunctional stabilizers formed by a small molecule recruiter of a deubiquitinase (DUB) bound to a ligand targeting the POI that needs to be stabilized, have recently emerged as new therapeutic strategies [82,83]. Their mode of action is summarized in Figure 8.

In 2022, Henning et al. proposed two molecules for targeted protein stabilization. The first molecule prevented the degradation of a mutated form of CFTR (ΔF508-CFTR), a common cystic fibrosis (CF) mutation, which is unstable and therefore, leads to rapid polyubiquitination and degradation of the protein, causing the phenotype observed in this disease. To stabilize this mutated form of CFTR, the authors used lumacaftor, a drug with affinity to ΔF508-CFTR used in CF, bound to the ligand EN523, a recruiter of the ubiquitin-specific deubiquitinase OTUB1. As proteasome-mediated degradation of the mutated-CFTR was hampered, human CF bronchial epithelial cells treated with this DUBTAC showed higher levels of CFTR [83].

The second DUBTAC designed by this group aimed to prevent ubiquitin-dependent proteasomal degradation of the tumor suppressor kinase WEE1. This DUBTAC, formed by the WEE1 inhibitor AZD1775 and the OTUB1 recruiter EN523, led to a stabilization of WEE1 similar to that observed upon treatment with the proteasome inhibitor bortezomib in the hepatoma cell line HEP3B [83].

In contrast to all the above mentioned TACnologies, which have shown their potential to tackle cancer by degrading the POI when it is overexpressed, DUBTACs can act where clinical consequences of the disease are due to the loss of the POI. Despite their potential, different considerations should be taken into account when designing DUBTACs, such as ligand length, protein stabilization kinetics, bioavailability, or mechanism of action. In addition, new targets that could benefit from this mode of action need to be identified [82,83].

## 8. Conclusions and Future Perspectives

TPD has been revealed as a promising new approach to cancer therapy that involves several therapeutic approximations; therefore, there is growing interest in the scientific community for its future clinical application, with an increasing number of authors discussing the prospects of this technology [29,84,85,86,87]. Currently, this approach has the potential to be highly effective and selective, as it can selectively target the proteins that drive cancer while leaving normal cells unharmed; even those classically classified as undruggable targets, which comprise the vast majority of the proteins encoded by our genome, including transcription factors [88]. Furthermore, TPD could overcome several limitations of current therapies such as drug-resistance phenomena and metastasis, describing these therapeutic agents as having potential roles in the future trends of cancer treatment and offering a platform to design personalized medicine.

Given the investment in the research of TPD that has been made in this century, there is little doubt that it will eventually become an important therapeutic modality. Despite their potential to become key treatment techniques, TPD strategies still have two challenges to overcome: delivery and dosage. To translate these technologies from bench to bedside, new delivery systems, such as those based on nanoparticles, need to be perfected. These nanosystems could not only improve the safety and the therapeutic efficacy of TPD by modulating systemic biodistribution and increasing the degrader molecules but also control the timing and localization of degradation [89].

In summary, TAC-based strategies, such as PROTAC, PHOTAC, CLIPTAC, AUTAC, ATTEC, LYTAC, DUBTAC, and all those to be developed in the coming years, are uniquely positioned to succeed in the treatment of hard-to-drug proteins. Using nanomedicine to improve their efficacy and reduce their side effects, these TACnologies are expected to the clinical applications within twenty years.

## Figures and Tables

**Table 1:** Summary of targets and pathways employed by current TACnologies.

Drug Type	Target	Pathway
AUTAC	Intracellular proteins and damaged organelles	Autophagy-lysosome
ATTEC	Intracellular proteins and non-proteic substrates	Autophagy-lysosomes
LYTAC	Extracellular and transmembrane proteins	Endosome-lysosome
PROTAC	Intracellular proteins	Ubiquitin-proteasome
Trivalent PROTAC	Intracellular proteins	Ubiquitin-proteasome
PHOTAC	Variety of targets: including BRD2-4 and FKBP12	Ubiquitin-proteasome
CLIPTAC	Intracellular proteins	Ubiquitin-proteasome
DUBTAC	Intracellular proteins	Ubiquitin-proteasome

AUTAC: AUtophagy TArgeting Chimeras; ATTEC: AuTophagosome TEthering Compounds; LYTAC: LYsosome-TArgeting Chimeras; PROTAC: PROteolysis TArgeting Chimeras; PHOTAC: PHOtochemically TArgeting Chimeras; CLIPTAC: CLIck-formed Proteolysis TArgeting Chimeras; DUBTAC: DeUBiquitinase-TArgeting Chimeras.

**Table 2:** Examples of uses of PROTAC-based TPD technology.

Drug Degrader	Compound	Ligase	Target	Reference
Heterofunctional PROTAC	KRIBB11	Pomalidomide	HSF1	[17]
	A-1155463	VHL	Bcl-xL	[18]
	AZD1775	VHL, CRBN	Wee1	[19]
	BI 1701963 (Phase I)	VHL	SOS1	[20]
	ARV-110 (Phase I)	CRBN	AR	[21]
	ARV-471 (Phase II)	CRBN	ER	[21]
PHOTAC	Opto-pomalidomide	CRBN	IKZF1/3	[22]
	Opto-dBET1	CRBN	BRD4	[23]
	Opto-dALK	CRBN	EML-ALK	[24]
	PHOTAC-II-5	CRBN	FKBP12	[25]
	PHOTAC-II-6	CRBN	FKBP12	[25]
Trivalent PROTAC	VZ185	CRBN	BRD9	[26]
			BRD7	
CLIPTAC	Tetrazine-tagged thalidomide	CRBN	BRD4 ERK1/2	[27]

AR, androgen receptor; Bcl-Xl, B cell lymphoma-extra large; BRD4, bromodomain-containing protein, 4; BRD7, bromodomain-containing protein, 7; BRD9, bromodomain-containing protein, 9; CLIPTAC, CLIck-formed Proteolysis TArgeting Chimeras; CRBN, cereblon; ER, estrogen receptor; ERK1/2, extracellular signal-regulated protein kinase; FKBP12, FK506-binding protein 12, HSF1, heat shock transcription factor 1; IKZF1/3, IKAROS family zinc finger 1/3; PHOTAC, PHOtochemically TArgeting Chimeras; PROTAC, PROteolysis TArgeting Chimeras; SOS1, Son of Sevenless Homologue 1; VHL, von Hippel–Lindau.

**Table 3:** Clinical trials registered using TPD.

Degrader	Type	Company	Target	E3 Ligase	Phase	Tumor Type	Identifier
ARV-110	Heterobifunctional	Arvinas	AR	CRBN	I	Prostate cancer	NCT03888612
ARV-471	Heterobifunctional	Arvinas, Pfizer	ER alpha	CRBN	II	Prostate cancer Breast cancer (ER+/HER2−)	NCT04072952
ARV-766	Heterobifunctional	Arvinas	AR	CRBN	I	Prostate cancer	NTC05067140
AC682	Heterobifunctional	Accutar Biotech	ER	CRBN	I	Locally Advanced or Metastatic ER+ Breast Cancer	NCT05080842
AR-LDD (CC-94676)	Heterobifunctional	Bristol Myers Squibb	AR	CRBN	I	Metastatic Castration-Resistant Prostate Cancer	NCT04428788
DT2216	Heterobifunctional	Dialectic	BCL-XL	VHL	I	Solid tumor Hematologic malignancy	NCT04886622
FHD-609	Heterobifunctional	Foghorn Therapeutics	BRD9	Undisclosed	I	Advanced Synovial Sarcoma or Advanced SMARCB1-Loss Tumors	NCT04965753
KT-333	Heterobifunctional	Kymera	STAT3	Undisclosed	I	Refractory Lymphoma Large Granular Lymphocytic Leukemia Solid Tumors	NCT05225584
KT-413	Heterobifunctional	Kymera	IRAK4	Undisclosed	I	Relapsed or refractory diffuse large B-cell lymphoma Marginal zone lymphoma Follicular lymphoma Primary central nervous system lymphoma Waldenstrom macroglobulinemia Nodular lymphocyte-predominant Hodgkin lymphoma.	NA
KT-474	Heterobifunctional	Kymera	IRAK4	Undisclosed	I	Healthy volunteers Atopic dermatitis Hidradenitis Suppurativa	NCT04772885
NX-2127	Heterobifunctional	Nurix Therapeutics	BTK	CRBN	I	Chronic lymphocytic leukemia Small lymphocytic lymphoma Mantle cell lymphoma Marginal zone lymphoma Waldenstrom macroglobulinemia Follicular lymphoma Diffuse Large B-cell Lymphoma	NCT04830137
NX-5948	Molecular glue	Nurix Therapeutics	BTK	CRBN	I	Hematological malignancies	Enter in late 2021
CFT7455	Molecular glue	C4 Therapeutics	IKZF 1/3	CRBN	I/II	Multiple myeloma and non-Hodgkin’s lymphomas	NCT04756726
CC92480	Molecular glue	Bristol-Myers Squibb	IKZF 1/3	CRBN	II	Multiple myeloma	NCT03989414
CC9982	Molecular glue	Bristol-Myers Squibb	IKZF 1/3	CRBN	II	Lymphoma non-Hodgkin’s Lymphoma large B-cell diffuse Lymphoma follicular	NCT03310619
CFT8634	Heterobifunctional	C4 Therapeutics	BRD9	CRBN	I/II	Synovial Sarcoma SMARCB1-Null Tumors	NCT05355753
CFT8919	Heterobifunctional	C4 Therapeutics	EGFR	CRBN		Non-small cell lung cancer	NA
DT2216	Heterobifunctional	Dialectic therapeutics	Bcl-Xl	VHL	I	Solid tumor Hematologic malignancy	NCT04886622
BGB-16673	Heterobifunctional	BeiGene	BTK	Undisclosed	I	B-cell malignancy marginal zone lymphoma Follicular lymphoma non-Hodgkin’s Lymphoma Waldenström macroglobulinemia	NCT05006716
FHD-609	Heterobifunctional	Foghorn Therapeutics Inc	BRD9	Undisclosed	I	Advanced synovial sarcoma	NCT04965753
CC220	Molecular glue	Bristol-Myers Squibb	IKZF1/3	CRBN	II	Multiple Myeloma	NCT02773030
CC90009	Molecular glue	Bristol-Myers Squibb	GSPT1	CRBN	II	Acute myeloid leukemia	NCT02848001 NCT04336982
CC99282	Molecular glue	Bristol-Myers Squibb	IKZF1/3	CRBN	I	Chronic myeloid leukemia, and non-Hodgkin’s lymphoma	NCT04434196 NCT03930953
CFT7455	Molecular glue	C4 Therapeutics	IKZF1/3	CRBN	I	Multiple Myeloma	NCT04756726
DKY709	Molecular glue	Novartis	Helios	CRBN	I	Solid tumors (Non-small-cell lung carcinoma)	NCT03891953

AR, androgen receptor; Bcl-Xl, B-cell lymphoma, extra large; BRD9, bromodomain-containing protein; 9; BTK, Bruton’s tyrosine kinase; CRBN, cereblon; GSPT1, G1 to S phase transition 1; EGFR, epidermal growth factor receptor; ER, estrogen receptor; IKZF1, IKAROS family zinc finger 1; IRAK4, interleukin-1 receptor-associated kinase 4; STAT3, signal transducer and activator of transcription 3; VHL, von Hippel–Lindau.

## Data Availability

Not applicable.
